# Oral immunotherapy for Immunoglobulin E‐mediated cow's milk allergy in children: A systematic review and meta analysis

**DOI:** 10.1002/iid3.704

**Published:** 2022-09-15

**Authors:** Lujing Tang, Yu Yu, Xiangyuan Pu, Jie Chen

**Affiliations:** ^1^ Department of Gastroenterology, The Children's Hospital, Zhejiang University School of Medicine National Clinical Research Center for Child Health Hangzhou China; ^2^ Department of Cardiology First Affiliated Hospital of Zhejiang University School of Medicine Hangzhou China

**Keywords:** children, cow's milk allergy, desensitization, meta‐analysis, oral immunotherapy

## Abstract

**Backgound:**

Cow's milk allergy (CMA) is the most common allergy in infants that decreases the quality of life of patients and their families. Standard treatment for CMA is the strict avoidance of milk; new treatment strategies such as oral immunotherapy (OIT) have been sought for patients with CMA. We aimed to assess the clinical efficacy and safety of OIT in the treatment of children with immunoglobulin E‐mediated CMA (IMCMA).

**Methods:**

We searched all randomized controlled trials in which OIT is used to treat children with IMCMA from five international electronic databases. We estimated a pooled risk ratio (RR) for each outcome using a Mantel–Haenzel fixed‐effects model if statistical heterogeneity was low.

**Results:**

Eleven studies were chosen for meta‐analysis, including a total of 469 children (242 OITs, 227 controls). One hundred and seventy‐six patients (72.7%) in the OIT were desensitized compared with 49 patients (21.6%) in the control group (RR: 7.35, 95% confidence interval (CI): 2.82–19.13, *p* < .0001). The desensitization effect of OIT was particularly significant in children over 3 years old (RR: 18.05, 95% CI: 6.48–50.26, *p* < .00001). Although adverse effects were common, they usually involved mild reactions, but epinephrine use was more common in the OIT group (RR: 7.69, 95% CI: 2.16–27.33, *p* < .002).

**Conclusion:**

OIT can lead to desensitization in the majority of individuals with IMCMA, especially in patients over 3 years old. A major problem of OIT is the frequency of adverse events, although most are mild. OIT may be an alternative treatment in the future.

## Graphical abstract

We conduct a latest meta‐analysis; the analyses show that oral immunotherapy (OIT) is effective for immunoglobulin E‐mediated cow's milk allergy in children, especially in patients older than 3 years. OIT can cause adverse actions but most are mild‐to‐moderate.

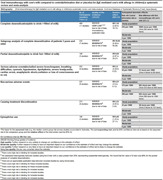

## 1 INTRODUCTION

Cow's milk allergy (CMA) is defined as a reproducible adverse reaction to cow milk (CM) protein mediated by an immunologic mechanism, involving immunoglobulin E (IgE)‐mediated, non‐IgE‐mediated, or mixed mechanisms.^1^ IgE‐mediated reactions are the commonest reactions, often occurring rapidly, typically within minutes to 2 h following the ingestion of small amounts of CM.^2^ Presentation varies in severity ranging from mild symptoms to rarely, life‐threatening anaphylaxis. Many children with CMA improve before school age, but in some cases it persists even into adulthood.^3^, ^4^ The current standard treatment for CMA is strict avoidance and emergency treatment of severe adverse reactions. However, milk is the main food for infants and is common in our life; it is difficult to avoid completely. Moreover, accidental exposure to CM can be potentially life‐threatening and has a major impact on quality of life (QoL).^5^ Strict avoidance has negative consequences in patients such as a risk of poor nutrition, increased levels of anxiety, and possible unjustified restrictions on further foods, with an increased immunological risk of nonacquiring tolerance.^6^ Therefore, it needs to find some new treatments, such as oral immunotherapy (OIT). OIT is an emerging approach to the treatment of patients with IgE‐mediated CMA (IMCMA).^1^, ^7^ OIT may increase the amount of food that the patient can tolerate, preventing allergic symptoms and reducing the risk of potentially life‐threatening allergic reactions. Thus, OIT may be potentially a curative therapy for IMCMA. Many studies have shown the efficacy of OIT in desensitization(an increased reaction threshold to a food allergen while receiving active therapy and might equate to protection from accidental ingestion) and some of them in sustained unresponsiveness (a lack of clinical reaction to a food allergen after active therapy has been discontinued for a period of time).^8^ However, there is an ongoing debate about the safety of OIT.^9^, ^10^ Because of the increasing interest in this topic and emerging studies, it is important to provide an up‐to‐date systematic review with ongoing updates. The main objective of this meta‐analysis is to assess the clinical efficacy and safety of OIT in children with IMCMA compared with placebo treatment or milk avoidance.

## 2 METHODS

### 2.1 Criteria for considering studies

Only randomized controlled trials (RCTs) were considered for inclusion, either blinded or open trial design. Studies were with no language restriction. The study population comprised children aged 0–18 years with IMCMA. We divided patients into two groups: a control or placebo group, in which children were treated with a milk‐avoidance diet or placebo, and an active group, in which children received milk OITs. Milk OITs administered by any protocol and OITs with other adjuvant treatments were included, a subgroup analysis of OITs with adjuvant treatment was conducted if possible. Patients with non‐IgE‐mediated adverse reactions to CM protein were excluded. Studies of other immunotherapies such as sublingual immunotherapy, subcutaneous immunotherapy, and epicutaneous immunotherapy were all excluded.

### 2.2 Outcome measures

#### 2.2.1 Primary outcomes

The primary outcome was successful desensitization: the ability to ingest a serving of CM (the minimal dose varies in each study) without adverse reactions while on therapy or continued daily ingestion.

#### 2.2.2 Secondary outcomes

1) Sustained unresponsiveness: Ability to ingest a serving of CM without adverse reactions after 4 weeks, or more, of stopping treatment.^11^
2) Partial desensitization: Ability to ingest a partial serving of CM without adverse reactions (the dose varies according to the different definitions in each study).^11^
3) Adverse events during OIT (serious adverse events include severe bronchospasm, breathing difficulties, cyanosis, hypotension, dysrhythmia, severe bradycardia, cardiac arrest, anaphylactic shock, confusion or loss of consciousness, and so on; nonserious adverse events mainly include mild and moderate skin symptoms, and gastrointestinal and respiratory symptoms).
4) Change in skin prick test (SPT) size, specific IgE level, and specific IgG4 level. Data were analyzed on an intention‐to‐treat (ITT) basis whenever possible.
5) Subgroup of the effect of adjuvant treatments such as OIT with omalizumab (OMB) was analyzed if possible. OMB is a humanized, monoclonal anti‐IgE antibody.

### 2.3 Electronic search methods

We performed a systematic search with no language restrictions of the following bibliographic databases: PubMed, Medline, Embase, BIOSIS citation index, and the Cochrane Library. The search was up‐to‐date as of April 30, 2021. In addition, we reviewed the references of the articles included to identify potentially relevant citations. A search was conducted including the following terms:

1. milk allergy/.
2. immune tolerance/.
3. immunotherapy/.
4. desensitization, immunologic/.
5. Remission Induction/.
6. desensiti*.tw.
7. immunotherapy.tw.
8. (oral adj3 (toleran* or induc*)).tw.
9. or/2‐8.
10. 1 and 9.

### 2.4 Data collection and analysis

Titles and abstracts of the records retrieved were examined by one reviewer and irrelevant records excluded. Subsequently, two reviewers evaluated full‐text records of all potentially eligible studies based on eligibility criteria and filtered out studies for this meta‐analysis. We developed a standardized data extraction form to extract study characteristics, then two reviewers (Lujing Tang and Yu Yu) extracted data about trial characteristics (setting, milk oral immunotherapy regimen, and eligibility criteria), methodological quality, participants, and outcomes of interest. Disagreements between reviewers were resolved with discussion.

We assessed the risk of bias of the included studies based on the criteria established by the Cochrane Handbook for Systematic Reviews of Interventions.^12^ In the meta‐analysis of RCTs, dichotomous outcomes were expressed as a risk ratio (RR) with 95% confidence intervals (CIs). Data were analyzed on an ITT basis whenever possible. All analyses were performed by Review Manager Version 5.3. We planned to perform subgroup analysis according to the patients' age (3 years and older). Sensitivity analyses were conducted to determine the influence of studies with a high risk of bias on the meta‐analysis.

### 2.5 Assessment of heterogeneity and reporting biases

We assumed that there would be clinical heterogeneity in the studies, including different ages of the study population and differences in the immunotherapy protocols. We assessed heterogeneity between studies using the *I* ^2^ test with a value >50% representing substantial heterogeneity. We estimated a pooled RR using a Mantel–Haenzel fixed‐effect model if *I* ^2^ test ≤50% or a random‐effect model if *I* ^2^ test >50%. Funnel plot was used to assess potential publication bias.

## 3 RESULTS

### 3.1 Included studies

**Figure 1:**
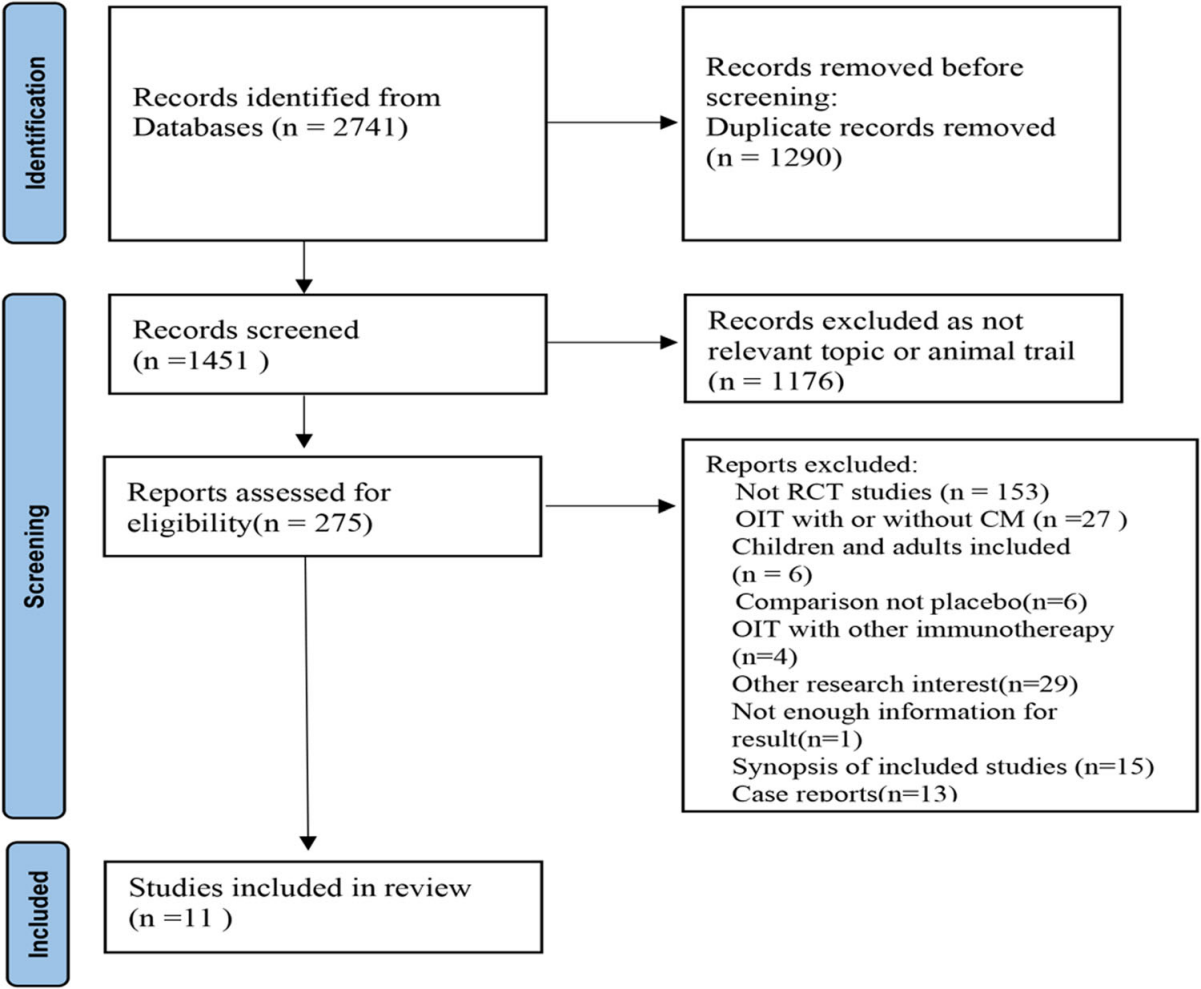
Results from searching for studies for inclusion in the review. CM, cow milk; OIT, oral immunotherapy; RCT, randomized controlled trial.

Our electronic search resulted in 2741 records, after removing duplications, screening titles, and abstracts remained, 275 records were screened again for eligibility by 2 reviewers independently. Discrepancies were resolved through discussion. After applying the inclusion and exclusion criteria, we selected 11 studies^13^, ^14^, ^15^, ^16^, ^17^, ^18^, ^19^, ^20^, ^21^, ^22^, ^23^ for this meta‐analysis (Figure 1). The characteristics of the included studies are summarized in Table 1.

**Table 1:** Characteristics of the included studies

	Number of patients	Age/years	Inclusion criteria	Exclusion criteria	Groups of treatment (*n*)		Dropouts	Maximum tolerated dose
Morisset 2007/France^13^	60	1.1–6.5	1. Positive CM‐SPT or sIgE 2. Positive labial or OFC 3. Recovery from symptoms after 3 weeks of CM avoidance	Patients who react with ≤60 ml of milk	OIT with CM (30)	Milk avoidance (30)	3	200 ml
Longo 2008/Italy^14^	60	5–17	1. History of severe allergic reactions 2. CM‐sIgE > 85 kUA/L 3. DBPCFC positive to ≤0.8 ml of whole milk	1. History of unreliable management of complications and treatments 2. Limited access to emergency facilities in the area where they lived 3. Uncontrolled asthma	OIT with CM (30)	Milk avoidance (30)	0	150 ml
Skripak 2008/USA^15^	20	6–17	1. Positive CM‐SPT 2. CM‐sIgE > 0.35 kUA/L 3. DBPCFC positive to dose ≤2.5 g of CMP	1. Anaphylaxis requiring hospitalization 2. History of intubation related to asthma 3. Current severe persistent asthma	OIT with CM (13)	Placebo (7)	1	500 mg
Pajno 2010/Italy^16^	30	4–10	1. Clinical history 2. Positive CM‐SPT or specific IgE 3. DBPCFC+	Sensitization to other foods	OIT with CM (15)	Soy milk (15)	3	200 ml
Martorell 2011/Spain^17^	60	2–3	1. Immediate‐type clinical symptoms 2. SPT ≥ 3 mm 3. CM‐sIgE > 0.35 kUA/L 4. DBPCFC+	1. History of anaphylactic shock 2. Non IgE‐mediated adverse reactions 3. Malignant or immunopathological diseases and/or immunodeficiencies 4. Therapy with immunosuppressors or β‐blockers 5. Contraindicating to epinephrine use	OIT with CM (30)	Milk avoidance (30)	5	200 ml
Salmivesi 2012/Finland^18^	28	6–14	1. SPT > 3 mm 2. CM‐sIgE > 3.5 kUA/L 3. Challenge test with CM‐positive or accidental exposure with a severe systemic reaction	Not mentioned	OIT with CM (18)	Placebo (10)	4	200 ml
Lee 2013/Korea^19^	31	0.5–1	1. A history of CMA 2. DBPCFC+	Not mentioned	OIT with CM (16)	Milk avoidance (15)	5	200 ml
Takahashi 2017/Japan^20^	16	6–14	1. Anaphylaxis history caused by CM or CM products by last 2 years 2. Sampson's symptoms score > Grade 2 associated with below 10 ml in OFC 3. DBPCFC+ 4. CM‐sIgE > 17.5 kUA/L 5. SPT ≥3 mm	1. Acute severe illness 2. Severe AD 3. Uncontrolled asthma	OIT with MHCM and OMB (10)	Milk avoidance (6)	0	200 ml
Esmaeilzadeh 2018/Iran^21^	84	0.5–3	1. Positive history of IgE‐mediated milk allergy 2. SPT ≥ 8 mm or sIgE‐CM >5 kUA/L (<2 years) and >15 kUA/L (>2 years old)	1. Nonspecific history 2. Negative SPT and undetectable sIgE levels 3. Unstable asthma, severe AD, and AR 4. EGE following milk ingestion 5. Recent reaction to BM products in past 6 months 6. Positive BM OFC	OIT with BM (42)	Milk avoidance (42)	0	240 ml or other products containing at 8–10 g skim milk
De Schryver 2019/Canada^22^	52	6–18	1. Clinical history of IgE‐CMA 2. SPT ≥ 3 mm and/or CMP‐IgE > 0.35 kU/L 3. SBPCFC+	1. Uncontrolled asthma 2. Malignancy 3. Autoimmune diseases 4. Severe primary and/or secondary immune deficiencies 5. Treatment with β‐blockers 6. Presence of cardiovascular disease or severe hypertension	OIT with CM (26)	Milk avoidance (26)	11	200 ml
Maeda 2021/Japan^23^	28	3–12	1. CM‐sIgE ≥ 0.7 UA/ml，DBPCFC positive 2. Clinical history 3. Able to make an emergency visit within 30 min 4. Written informed consent from the parents	1. History of life‐threatening anaphylactic shock 2. Uncontrolled asthma and AD 3. Allergic to other foods used in the DBPCFC test 4. Ineligible because of complications 5. Unable to obtain consent 6. Difficulty to withdraw drugs in food challenge test	OIT with CM (14)	Milk avoidance (14)	2	100 ml

Abbreviations: AD, atopic dermatitis; AR, allergic rhinitis; BM, baked milk; CM, cow's milk; CMA, cow's milk allergy; CMP, cow's milk protein; DBPCFC, double‐blind placebo‐controlled food challenge; EGE, eosinophil gastroenteropathy; MHCM, microwave‐heated cow milk; OFC, oral food challenge; OIT, oral immunotherapy; OMB, omalizumab; SBPCFC, single‐blind placebo‐controlled food challenge; SPT, skin prick test; sIgE, specific IgE.

These 11 studies were published between 2007 and 2021, a total of 469 children (242 OITs and 227 controls) were included, of which 234 patients (126 OITs and 108 controls) were older than 3 years, and a subgroup analysis was conducted for these patients. IMCMA was confirmed by a double‐blind placebo‐controlled food challenge (DBPCFC) in eight of the studies^14^, ^15^, ^16^, ^17^, ^18^, ^19^, ^20^, ^23^ and by a simple‐blind placebo‐controlled food challenge in two studies.^13^, ^22^ However, in the study of Esmaeilzadeh et al.,^21^ IMCMA was diagnosed by a history of immediate onset of symptoms after ingesting CM and positive SPT and/or IgE antibodies to CM. Eight studies used continued elimination diet as a control,^13^, ^14^, ^17^, ^19^, ^20^, ^21^, ^22^, ^23^ whereas the other two studies used a placebo control^15^, ^18^ and Pajno et al.^16^ used soy milk as a control. Most of the included studies used raw CM for OIT, but Esmaeilzadeh et al.^21^ used baked milk for OIT and Takahashi et al.^20^ combined OIT with OMB as the treatment group. The efficacy of desensitization was evaluated by identifying the maximum tolerated dose of milk in the individual studies, as follows: 240 ml^21^, 200 ml^13^, ^16^, ^17^, ^18^, ^19^, ^20^, ^22^, 150 ml^14^, 100 ml,^23^ and 500 mg.^15^ Four studies included patients younger than 3 years old^13^, ^17^, ^19^, ^20^ and two studies included only children with a history of severe anaphylaxis to milk,^14^, ^20^ whereas other three studies excluded such patients^15^, ^17^, ^23^ and the rest of the studies included patients with any degree of reaction. The OIT protocol was different in each study, most of them involved a build‐up phase in an institution (hospital, clinic, or research center) followed by periodic up‐dosing (either in a clinic or at home) and maintenance at home, but Salmivesi et al.^18^ conducted OIT trials in the outpatient clinic and Takahashi et al.^20^ did not illustrate this point.

### 3.2 Assessment of quality

**Figure 2:**
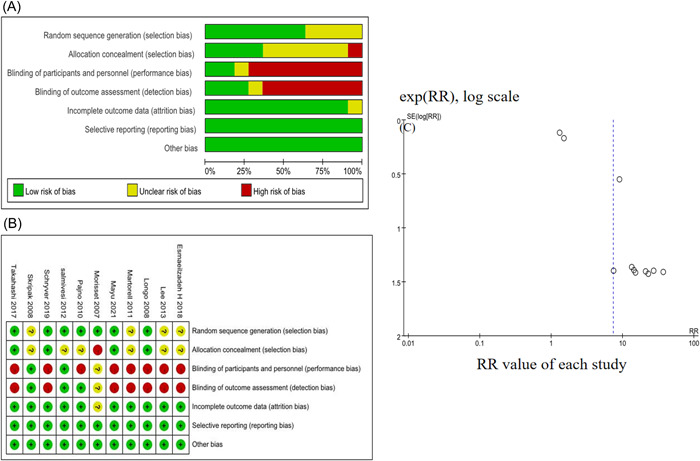
(A) The summary of risk of bias for all included studies. (B) Each risk of bias item for each included study. (C) Funnel plot of all included studies. RR, risk ratio.

Figure 2A,B presents the assessment of the risk of bias of the 11 included studies. There was an appreciable publication bias between the included studies by using funnel plots (Figure 2C).

### 3.3 Effect of interventions

#### 3.3.1 Efficacy

**Figure 3:**
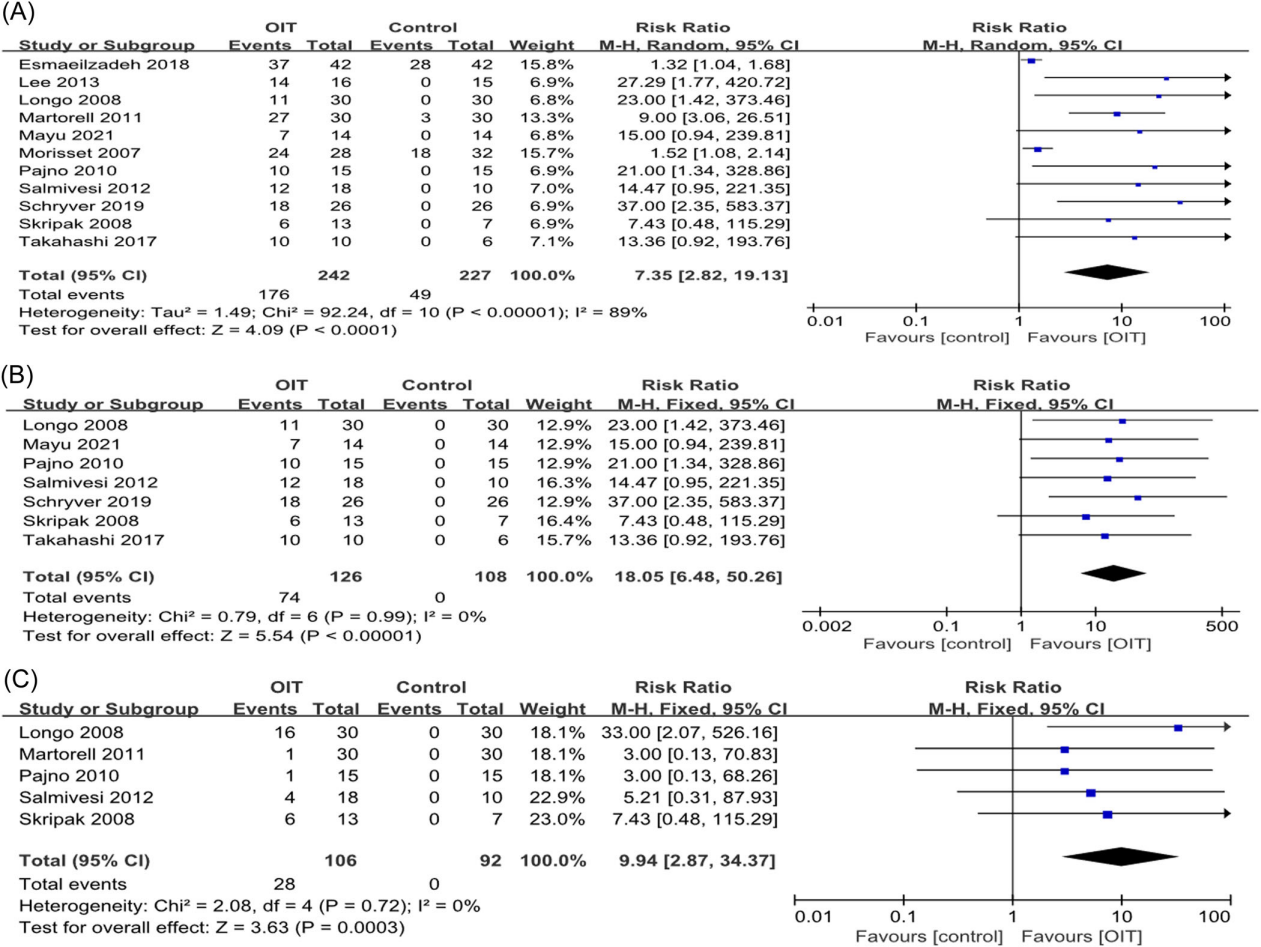
Efficacy of OIT. (A) Desensitization, (B) subgroup analysis of desensitization in children older than 3 years old, and (C) partial desensitization. CI, confidence interval; OIT, oral immunotherapy.

The major objective of our meta‐analysis was to determine the efficacy of OIT for IMCMA. All 11 studies described the desensitization of OIT and a total of 469 patients were quantitatively analyzed (242 OITs and 227 controls). Our meta‐analysis showed that 176 patients (72.7%) of the patients receiving OIT were able to be completely desensitize compared with 49 (21.6%) of the control group, with a pooled RR of 7.35 (95% CI: 2.82, 19.13; *p* < .0001; Figure 3A). After excluding patients younger than 3 years old, there was no heterogeneity between the rest of the studies. Therefore, we did a subgroup analysis for these patients over 3 years old and the results showed that 74 (58.7%) patients in the OIT group achieved desensitization, whereas there was no one in the control group, with a pooled RR of 18.05 (95% CI: 6.48, 50.26; *p* < .00001; Figure 3B). Because of obvious publication bias, we also performed a sensitivity analysis to ensure that any included study would not affect the overall results (Table 2). In addition, there were five studies that described the effect of OIT on partial desensitization. The definition of partial desensitization is not the same between studies. The analysis showed that the OIT group had a higher rate of partial desensitization than the control group (RR: 9.94, 95% CI: 2.8, 34.37; *p* = .0003; Figure 3C). There are only two studies reporting sustained unresponsiveness. Salmivesi et al.^18^ reported that among 28 patients, 23 and 22 were able to use significant amounts of CM 6–12 months and 3–3.5 years, respectively, after desensitization. Maeda et al.^23^ showed that seven in eight patients were able to continually ingest more than 100 ml of CM 2 years after the completion of the study.

**Table 2:** Sensitivity analysis

	RR [95%CI]	*I* ^2^	*p*
Exclude Morisset et al/2007^13^	10.92 [1.82,65.63]	91%	.009
Exclude Longo et al/2008^14^	5.57 [2.19,14.18]	88%	.0003
Exclude Skripak et al/2008^15^	6.29 [2.37,16.68]	89%	.0002
Exclude Pajno et al/2010^16^	5.62 [2.2,14,41]	88%	.0003
Exclude Martorell et al/2011^17^	5.52 [2.14,14.25]	86%	.0004
Exclude Salmivesi et al/2012^18^	5.84 [2.26,15.14]	89%	.0003
Exclude Lee et al/2013^19^	6.39 [2.49,16.42]	89%	.0001
Exclude Takahashi et al/2017^20^	5.9 [2.26,15.36]	89%	.0003
Exclude Esmaeilzadeh et al/2018^21^	10.73 [2.36,48.82]	86%	.002
Exclude De Schryver et al/2019^22^	5.18 [2.11,12.68]	87%	.0003
Exclude Maeda et al/2021^23^	5.87 [2.25,15.27]	89%	.0003

Abbreviations: CI, confidence interval; RR, risk ratio.

#### 3.3.2 Adverse events

**Figure 4:**
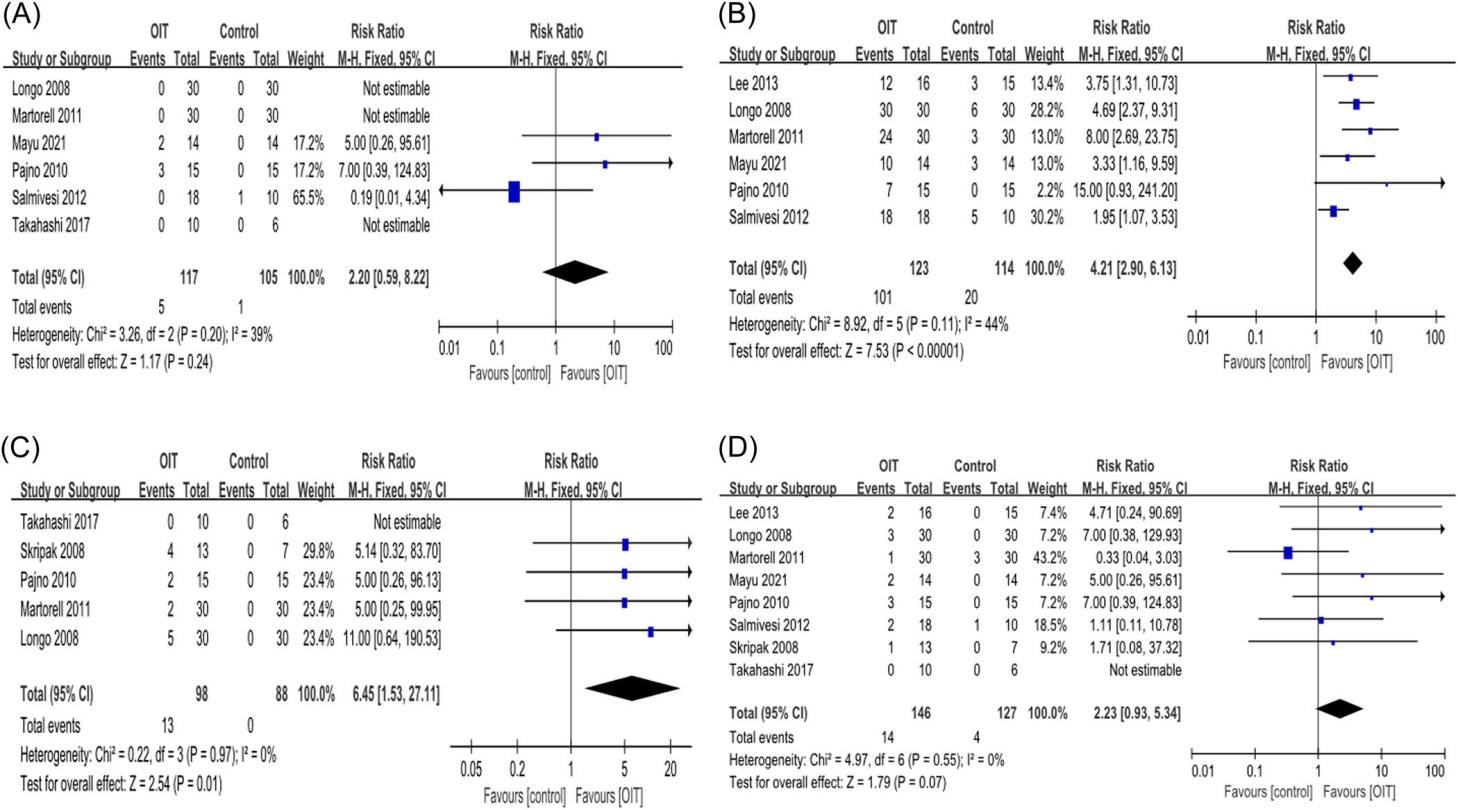
Adverse events of OIT. (A) Serious adverse events, (B) nonserious adverse events, (C) epinephrine use, (D) treatment discontinuation. CI, confidence interval; OIT, oral immunotherapy.

Six studies were included for analyzing the serious adverse events of OIT. There were only six patients together, who experienced serious adverse events, five from the OIT group and one from the control group, with a pooled RR of 2.2 (95% CI: 0.59, 8.22; *p* = .24; Figure 4A) and there is no statistical difference. In addition, there were also six studies describing nonserious adverse events of OIT, 82.1% (101/123) in the OIT group compared with 17.5% (20/114) in the control group; the RR value was 4.21 (95% CI: 2.9, 6.13; *p* < .00001; Figure 4B) and there is a statistical difference. We also analyzed epinephrine use and treatment discontinuation during OIT. There were, respectively, six and eight studies included and the RR value was 6.45 (95% CI: 1.53, 27.11; *p* = .01; Figure 4C) for epinephrine use and 2.23 (95% CI: 0.93, 5.34; *p* = .07; Figure 4D) for treatment discontinuation. There is a statistical difference for epinephrine use but not for treatment discontinuation.

#### 3.3.3 Immunological changes

We intended to analyze the immunological changes before and after the intervention, such as the change of CM‐specific IgE, anti‐casein IgE, anti‐β‐lactoglobulin IgE, α‐lactalbumin specific IgE, IgG4, and SPT size, but because of different expression in each study, some used mean value and some used median value, so it was impossible to do systemic analysis.

### 3.4 Other adjuvant therapies combined with OIT

We also hoped to analyze the efficacy of other adjuvant therapies combined with OIT, for example, OIT combined with OMB. Among our included studies, there was only one study associated with OMB. Takahashi et al.^20^ investigated the efficacy of OIT with OMB. In the treatment group, the patients accepted OMB from the beginning of the study every 2 to 4 weeks until 24 weeks, then OIT was started after the first 8 weeks of OMB treatment and was maintained for 32 weeks. At Week 32, all 10 OMB–OIT‐treated patients and none of the 6 untreated patients passed DBPCFC (*p* < .001). A significantly decreased SPT diameter was found in the OMB–OIT‐treated group (*p* < .05).

## 4 DISCUSSION

IMCMA is an increasing global health problem and proactive treatments are needed to reduce the burden of CMA. A significant amount of research have been directed at various forms of food immunotherapy, including oral, sublingual, and epicutaneous delivery routes. OIT is mostly used to treat peanut, milk, and egg allergy. As the OIT protocol used and the duration time are varied in each study, efforts have been done to improve the usefulness of the technique and establish protocols for more widespread use.

We conducted this systematic analysis of all RCTs in which OIT was used as a treatment for IMCMA. After comparing with the control group, our analysis shows that about three‐quarters receiving OIT were completely desensitized. OIT with CM may be an effective and safe alternative therapy for children with IMCMA. The conclusions on the efficacy of OIT are similar to other studies.^7^, ^11^, ^24^, ^25^, ^26^ The main benefit of OIT is the ability to accidentally consume even a small amount of CM (e.g., 5 ml) or milk products without a reaction. Thus, it is necessary to evaluate the effect of OIT on partial desensitization. According to our analysis, one‐quarters in the OIT group got partially desensitized, comparing with no one in the control group. Although there was heterogeneity among the included studies, it may be associated with patients' age, small patients' number, different protocol, and so on; thus, we did a subgroup analysis and sensitivity analysis, and obtained similar conclusions. We intended to analyze the effect of OIT on sustained unresponsiveness but there are not enough studies included for meta‐analysis. There is no insufficient evidence to draw conclusions and more high‐quality trials are needed to explore the impacts of OIT on sustained unresponsiveness.

Adverse events during OIT are common, whereas most are mild–moderate and easily managed. Our analysis showed that there were only six patients with serious adverse events (five in the OIT group and one in the control group) and none was life‐threatening. There were only 13% and 9.6% patients needing epinephrine use and treatment discontinuation, respectively, although the rate was higher than the control group. In general, OIT is usually well tolerated. We also planned to analyze OIT on the impact of QoL but there were no RCTs. A pilot study showed that OIT may improve the QoL in emotional impact, food anxiety, social limitation, and dietary limitation domains, particularly in children over 4 years old.^27^ Other two studies concluded that the total Food Allergy Quality of Life Questionnaire Parent Form scores and the total Food Allergy Quality of Life Questionnaire Children Form scores were both improved.^28^, ^29^

To decrease the adverse actions of OIT, many therapies were studied. Baked milk is likely to be hypoallergenic in part because of changes in the higher‐order structure of conformational epitopes. Some studies^21^, ^30^ showed that OIT with baked milk maybe effective, but the World Allergy Organization guideline^7^ suggests that clinicians do not use OIT with baked CM in people with IMCMA, who do not tolerate unheated and baked milk. Another popular adjuvant regimen is the use of biologics. There is no currently United States Food and Drug Administration‐approved biologic therapy for use in food allergy. OMB has been studied as monotherapy and as an adjuvant therapy in the treatment of food allergies, in conjunction with OIT. OMB binds to the heavy chain constant CH3 domain of the free IgE molecule and prevents IgE from binding to FceRI effector cells. There were several studies of OMB with milk OIT,^20^, ^31^, ^32^, ^33^, ^34^ including two RCTs (one compared with milk avoidance and the other compared with OIT alone). OIT with OMB may allow a shorter build‐up phase or higher median tolerated dose, but adverse reactions, including the need for epinephrine, still occurred. Therefore, it needs more RCTs to examine the efficacy and safety of OMB. Other biologics (TNX‐901, Mepolizumab, Bbenralizumab, Reslizumab, Dupilumab, Ligelizumab, Ibrutinib, Etokimab, and so on) have been used in other atopic diseases and/or food allergy, but not used in CM allergy.^35^

Desensitization to CM through immunotherapy has been associated with a decrease in CM‐sIgE levels and an elevation in sIgG4 levels,^36^, ^37^, ^38^, ^39^ suggesting that upregulation of allergen‐specific IgG4 responses may be an important event in CM‐specific immunotherapy. We intended to analyze the immunological changes before and after the intervention but most of the included studies described IgE values differently, some in mean and some in the median, so it was impossible to do a combined analysis. On the other hand, only four studies described IgG4 level changes,^15^, ^16^, ^19^, ^20^ whereas the values were expressed inconsistently.

**Figure 5:**
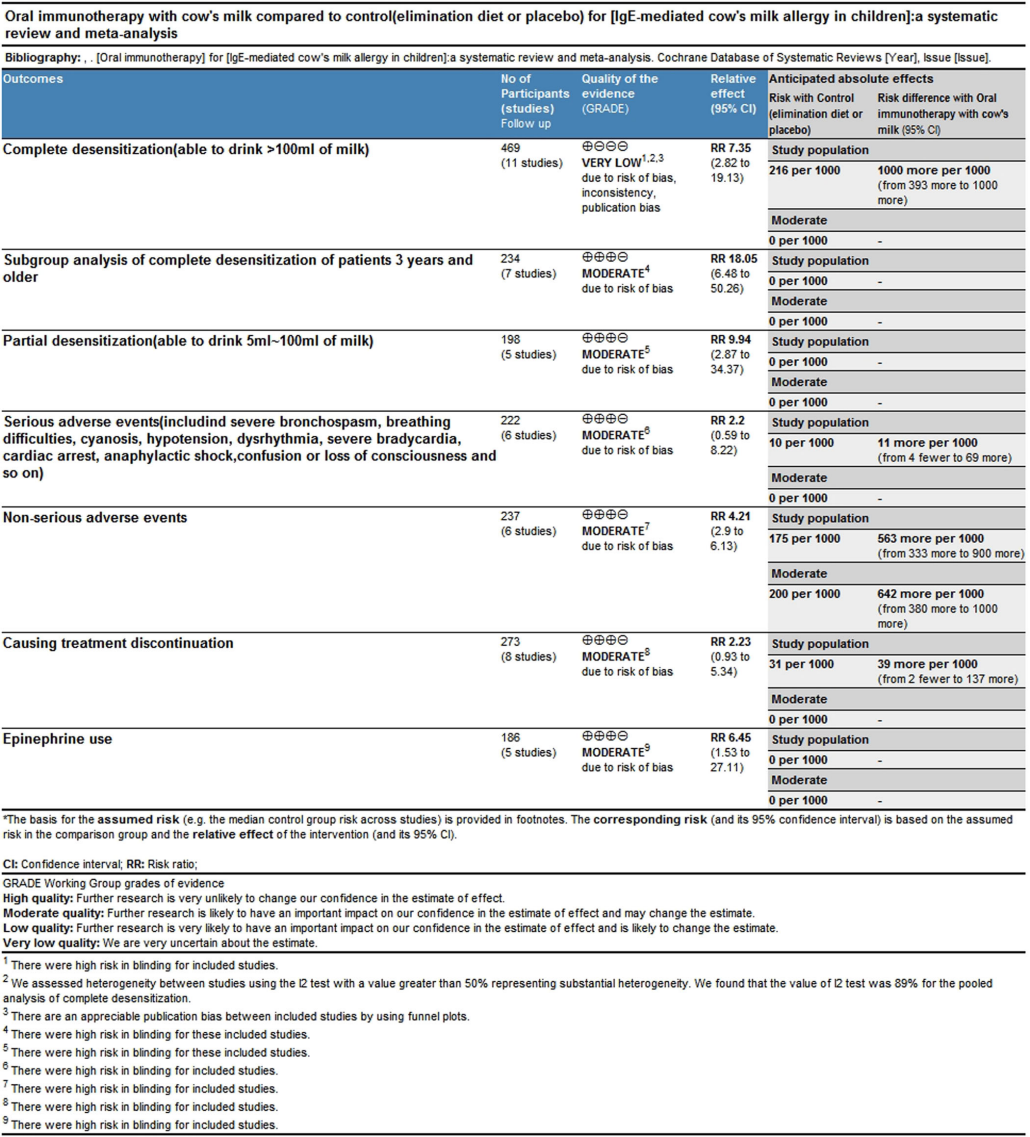
Summary of findings table for a question—Should oral immunotherapy be used in children with IgE‐mediated cow's milk allergy

The evidence supporting the use of OIT in IMCMA, however, is of very low quality because of a high likelihood of bias and heterogeneity, but it is of moderate quality for children who are older than 3 years (Figure 5). Other limitations of our study include that we can not perform a meta analysis of safety or changes in skin reactivity and experimental results, owing to differences in the presentation of results. However, this limitation can be corrected through consensus on measuring these variables in future OIT studies. Furthermore, we do not conduct analysis of sustained unresponsiveness and OIT with adjuvant therapy, because there are no enough studies.

## 5 CONCLUSION

Our meta‐analysis showed that OIT maybe effective for children with IMCMA, especially for children older than 3 years, and the adverse events during OIT cannot be ignored, but most of adverse actions are mild to moderate and epinephrine using is not uncommon. OIT may be an alternative treatment for CMA, but it needs more high‐quality RCTs to find out a standard protocol of OIT and to explore the impacts on QoL, sustained unresponsiveness, and adding with biologics. Clinicians and families will need to weigh up the benefits and harms when considering whether immunotherapy is appropriate for individuals.

## CONFLICT OF INTEREST

The authors declare no conflict of interests.

## ETHICAL APPROVAL

All analyses were based on previously published studies; thus, no ethical approval and patient consent are required.
